# PCAO2: an ontology for integration of prostate cancer associated genotypic, phenotypic and lifestyle data

**DOI:** 10.1093/bib/bbae136

**Published:** 2024-03-31

**Authors:** Chunjiang Yu, Hui Zong, Yalan Chen, Yibin Zhou, Xingyun Liu, Yuxin Lin, Jiakun Li, Xiaonan Zheng, Hua Min, Bairong Shen

**Affiliations:** Department of Urology and Institutes for Systems Genetics, Frontiers Science Center for Disease-related Molecular Network, West China Hospital, Sichuan University, Chengdu, 610041, China; School of Artificial Intelligence, Suzhou Industrial Park Institute of Services Outsourcing, Suzhou, 215123, China; Center for Systems Biology, Soochow University, Suzhou, 215006, China; Department of Urology and Institutes for Systems Genetics, Frontiers Science Center for Disease-related Molecular Network, West China Hospital, Sichuan University, Chengdu, 610041, China; Department of Urology and Institutes for Systems Genetics, Frontiers Science Center for Disease-related Molecular Network, West China Hospital, Sichuan University, Chengdu, 610041, China; Center for Systems Biology, Soochow University, Suzhou, 215006, China; Department of Medical Informatics, School of Medicine, Nantong University, Nantong, 226001, China; Department of Urology, The Second Affiliated Hospital of Soochow University, Suzhou, 215011, China; Department of Urology and Institutes for Systems Genetics, Frontiers Science Center for Disease-related Molecular Network, West China Hospital, Sichuan University, Chengdu, 610041, China; Department of Urology, The First Affiliated Hospital of Soochow University, Suzhou, 215000, China; Department of Urology and Institutes for Systems Genetics, Frontiers Science Center for Disease-related Molecular Network, West China Hospital, Sichuan University, Chengdu, 610041, China; Department of Urology and Institutes for Systems Genetics, Frontiers Science Center for Disease-related Molecular Network, West China Hospital, Sichuan University, Chengdu, 610041, China; Department of Health Administration and Policy, George Mason University, Fairfax, VA, USA; Department of Urology and Institutes for Systems Genetics, Frontiers Science Center for Disease-related Molecular Network, West China Hospital, Sichuan University, Chengdu, 610041, China

**Keywords:** prostate cancer, ontology, knowledge representation, knowledge graph, deep phenotyping

## Abstract

Disease ontologies facilitate the semantic organization and representation of domain-specific knowledge. In the case of prostate cancer (PCa), large volumes of research results and clinical data have been accumulated and needed to be standardized for sharing and translational researches. A formal representation of PCa-associated knowledge will be essential to the diverse data standardization, data sharing and the future knowledge graph extraction, deep phenotyping and explainable artificial intelligence developing. In this study, we constructed an updated PCa ontology (PCAO2) based on the ontology development life cycle. An online information retrieval system was designed to ensure the usability of the ontology. The PCAO2 with a subclass-based taxonomic hierarchy covers the major biomedical concepts for PCa-associated genotypic, phenotypic and lifestyle data. The current version of the PCAO2 contains 633 concepts organized under three biomedical viewpoints, namely, epidemiology, diagnosis and treatment. These concepts are enriched by the addition of definition, synonym, relationship and reference. For the precision diagnosis and treatment, the PCa-associated genes and lifestyles are integrated in the viewpoint of epidemiological aspects of PCa. PCAO2 provides a standardized and systematized semantic framework for studying large amounts of heterogeneous PCa data and knowledge, which can be further, edited and enriched by the scientific community. The PCAO2 is freely available at https://bioportal.bioontology.org/ontologies/PCAO, http://pcaontology.net/ and http://pcaontology.net/mobile/.

## INTRODUCTION

Precision medicine is a novel medical paradigm that revolves around personalized diagnosis, treatment and healthcare. It has emerged as a result of the rapid advancements in genome sequencing technology and its application in the realm of biological information and big data science [1, 2]. Comparing to the evidence-based medicine approach, which relies on population-level validation and averaging, precision medicine relies on comprehensive genotypic, phenotypic and lifestyle data for personalized modeling and application [3–5]. Consequently, it becomes crucial to gather relevant genotypic, phenotypic and lifestyle data for each individual in order to accurately predict their health status. However, clinicians and researchers still face challenges in systematically collecting diverse disease-specific concepts, their synonyms and intricate relationships, combining their personal knowledge and experience. Despite the growing utilization of artificial intelligence (AI) in medicine, there remains a substantial need for a significant amount of labeled and structured data in order to facilitate modeling, pattern recognition and knowledge discovery. In order to address the inherent limitation of current AI models as black boxes, it becomes crucial to embed relationships between concepts or knowledge graphs. This enables the development of explainable AI (XAI) models that can be trusted by both patients and clinicians, promoting trustworthiness and transparency [6].

Ontology plays a crucial role in explicitly expressing knowledge, encoding semantics and fostering a shared understanding of knowledge within a problem domain for both humans and computers [7]. Its application in precision medicine is extensive [8]. Biomedical research objects, such as genes, proteins, drug targets, diseases and clinical data, can benefit from text mining, providing valuable reference information for biologists and medical researchers. However, a significant challenge in this type of research lies in effectively and efficiently utilizing AI methods to screen relevant literature from vast volumes of information, while also assessing the relationships between the study objectives described in the literature and related data. In this context, the establishment and enhancement of domain ontologies and controlled vocabularies serve as a crucial foundation for improving the performance of text knowledge mining and enabling automatic knowledge classification [9]. Researchers increasingly require the ability to access, interpret and analyze data from various biological literature and annotated resources in a unified manner. To address the integration of heterogeneous data, various technologies have been developed over the years, including the Open Database Connectivity (ODBC) standard, Extensible Markup Language (XML)based standards, web services, service-oriented architectures, data warehousing and database federation. However, these technologies primarily address the integration of heterogeneous data at the data level and not at the semantic level. Ontology-based approaches are well suited for overcoming heterogeneity and semantic conflicts during the integration of heterogeneous data [10]. A major obstacle preventing the widespread adoption of a clinical decision support system (CDSS) in clinical practice is the challenge of representing domain knowledge and patient data within a unified framework. A CDSS that leverages ontology to integrate domain knowledge and patient data tends to be more accurate and enjoy higher acceptance rates [11].

Prostate cancer (PCa) ranks as the second most common malignancy in men worldwide [12]. In the United States, its incidence surpasses that of lung cancer, making it a significant threat to male health [13, 14]. According to statistics from the American Cancer Society, the number of PCa patients in the United States during 2023 will be 288 300 [15]. The number of deaths due to PCa has decreased from 32 050 in 2010 to 27 540 in 2015 [16]. In Europe, there were approximately 382 000 new cases of PCa in 2008, accounting for 22% of all cancers in men [17]. While the incidence of PCa has historically been lower in China compared to Western countries, it has been steadily rising over the past decade, and the rate of increase has accelerated [18]. Since 2008, PCa has become the most prevalent tumor affecting the urinary system in China. According to the 2015 annual report from the National Central Cancer Registry of China, PCa had an incidence rate of 7.10 per 105 individuals in 2011, ranking it seventh among male malignancies. The mortality rate for PCa was 2.98 per 105 individuals, placing it ninth among all male malignancies [19].

Risk factors for PCa remain uncertain, but they encompass age, race, heredity and diet [20, 21]. The World Cancer Research Fund/American Institute for Cancer Research (WCRF/AICR) report ‘Food, nutrition, physical activity and the prevention of cancer: a global perspective (2014)’ highlights the growing significance of lifestyle and environmental factors in PCa occurrence. PCa primarily affects older men, with a median age of diagnosis at 72 years and a peak age range of 75–79 years [22]. In the United States, African Americans have the highest incidence and mortality rates of PCa [23]. Having a brother or father with PCa increases the risk two to three times compared to individuals without a family history of the disease [24].

The data and knowledge related to PCa are diverse, but they require standardization and structuring for effective knowledge discovery and explainable AI modeling. Among various research fields, the concept of ontology has gained widespread acceptance and development, serving as a formal representation of domain knowledge in biomedical informatics. Currently, numerous reusable ontologies have been established, including the Gene Ontology (GO), Disease Ontology (DO), Protein Ontology and Sequence Ontology. Specifically, GO is primarily utilized for annotating genes and their products in terms of biological function, biological process or subcellular component. Since its establishment in 1998, GO has rapidly emerged as a crucial method and tool for bioinformatics studies [25]. DO was constructed through the semantic integration of disease terminologies from various medical knowledge databases, including Medical Subject Headings (MeSH) [26], International Classification of Diseases (ICD), National Cancer Institute (NCI) Thesaurus [27], Systematized Nomenclature of Medicine-Clinical Terms (SNOMED CT) [28] and Online Mendelian Inheritance in Man (OMIM) [29]. Intricate logical relationships were established between these terminologies [30]. Currently, widely used biomedical ontologies such as HPO and DO include concepts pertaining to human phenotypes and diseases. These ontologies are high level and provide broad coverage, but they lack in-depth and comprehensive information from multi-source for specific diseases [31]. For researchers and clinicians seeking a comprehensive understanding of a specific disease, integrating information from different sources can be a time-consuming and challenging task. Moreover, the precision medicine requires an understanding of precision relationships between genotype and phenotype [4, 32]. Multiple well-established disease ontologies, including those for Parkinson’s disease [31] and Alzheimer’s disease [33], facilitate the standardization of diverse data and knowledge within their respective domains.

Currently, there is a lack of ontologies for integrating diverse data on specific cancers. An initial version of the PCa ontology (PCAO1) was proposed by one of our co-authors in 2009 [10]. However, it was not made publicly available and did not include genotypic and lifestyle data. In this study, we have developed an updated version of the PCa Ontology (PCAO2) to effectively represent, communicate and share knowledge on PCa in a unified and structured manner. The PCAO2 encompasses key concepts from three perspectives: epidemiology, diagnosis and treatment. Based on the precision medicine paradigm, we integrate genetic and epigenetic information, including PCa-related genes, lifestyles and environmental factors, to offer a comprehensive epidemiological understanding of PCa. Our objective is to create a formal representation of PCa concepts. This will facilitate diverse data standardization, data sharing and enable future extraction of knowledge graphs, deep phenotyping and the development of explainable AI.

## MATERIALS AND METHODS

**Figure 1:**
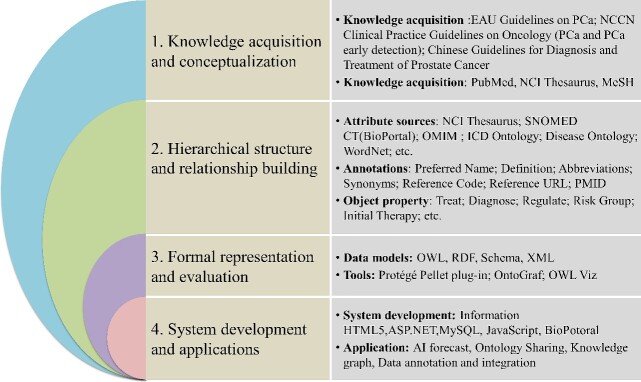
PCAO2 development process. The main process includes (1) knowledge acquisition and conceptualization; (2) hierarchical structure and relationship building; (3) formal representation and evaluation; and (4) system development and applications.

As shown in Figure 1, PCa knowledge was collected first from existing ontologies, literature, clinical guidelines, clinical database system and other resources. Subsequently, concepts pertaining to PCa were extracted from these knowledge sources. To annotate the extracted concepts, SNOMED CT, NCI Thesaurus, MeSH, Unified Medical Language System (UMLS), PCa-related guidelines and other resources were utilized. Under the supervision of experienced doctors, we classified concepts, restructured the hierarchical organization and established logical relationships. The PCAO2 was developed using the Ontology Web Language (OWL) format and the Protégé editor. Furthermore, an online information retrieval system was designed based on the PCAO2. The detailed process of PCAO2 development is explained in the following subsections.

### Knowledge acquisition and conceptualization

To compile a comprehensive collection of PCa terms and concepts, we extensively reviewed multiple knowledge sources. These included esteemed references like the European Association of Urology (EAU) Guidelines on PCa, National Comprehensive Cancer Network (NCCN) Clinical Practice Guidelines on Oncology (PCa and PCa early detection), NCCN Guidelines for Patients, China Guidelines for the Diagnosis and Treatment of Prostate Cancer, Optimal Care Pathway for Men with Prostate Cancer and Chinese Prostate Cancer Database. By focusing on the perspectives of epidemiology, diagnosis and treatment, we extracted relevant PCa-related concepts. We also utilized various online resources, including the NCI Thesaurus, SNOMED CT, MeSH, OMIM, ICD, DO, Foundational Model of Anatomy (FMA), online books, PubMed and Google, to expand the scope of concepts in describing the knowledge domains of PCa. To ensure standardization of lifestyle data, our previously developed PCa lifestyle ontology (PCLiON) was integrated into PCAO2 [34, 35]. Additionally, the GTR database served as the source for extracting genes associated with PCa [36]. These concepts were manually extracted to ensure accuracy. The concepts with same meaning have been standardized and merged.

### Hierarchical structure and relationship building

During concept collection, we also gathered hierarchical structures and logical relationships associated with the concepts. Each concept was annotated with its preferred name, definition, synonyms, abbreviations, reference code, reference Uniform Resource Locator (URL) and PubMed ID. It is important to note that the current version of PCAO2 doesn’t cover all concepts related to PCa. Therefore, we need to revise existing concepts, incorporate new knowledge and continue developing PCAO2 similar to other disease ontologies.

The expert panel’s validation of the ontological structure serves as an authentic evaluation of the disease ontology [37]. The PCAO2 underwent revisions by PCa experts, gradually forming the overall framework and details through multidisciplinary team meetings (Supplementary Text S1). They conducted a comprehensive review of the structure and relationships, confirming each concept and its interconnections, while also suggesting constructive amendments. Given the dynamic nature of PCa field research, the structure of the PCAO2 remains subject to future updates and adjustments.

### Formal representation and evaluation

In this study, Protégé was chosen as the construction tool due to its user-friendly interface, continuous quality enhancements, free access and robust functional extensibility. PCAO2 was built using the Protégé editor in OWL format. Classes were annotated with labels, definitions, references, synonyms, URLs and PubMed Identifiers (PMIDs). Axioms were used to establish logical relationships between class expressions, object properties were employed to connect pairs of individuals of classes and data properties were used to connect individuals of classes with literals.

We validated the quality of the PCAO2 through tool evaluation and domain expert evaluation. The reasoners Hermit [38] and Pellet [39] were utilized to check consistency, classify the ontology and compute inferred types [40]. OntoGraf was used to interactively navigating the relationships of the ontology [41]. Additionally, several domain experts were involved as evaluators to manually assess the accuracy, clarity and completeness of the ontology.

### System development and applications

An online information retrieval system was developed for PCAO2. The contents of PCAO2 are accessible via a web browser and are available in both English and Chinese. Mobile intelligent terminals, such as smartphones and tablets, are supported with dedicated functionality. Additionally, open data interfaces have been implemented to enable seamless integration with other applications (Supplementary Text S2).

## RESULTS

### Overview of PCAO2

The latest version of the PCAO2 contains 633 concepts and 2386 synonyms. The structural parameters for the PCAO2 are summarized in Table 1. The PCAO2 concepts were categorized into epidemiological, diagnostic and therapeutic viewpoints (Supplementary Text S3, Supplementary Figure S1). The maximum depth is 7, which includes the following levels: prostate cancer, therapeutic aspects of prostate cancer, other treatment, post-treatment quality of life in patients with localized prostate cancer, post-treatment hormonal therapy, side-effects of hormonal therapy, other systemic side-effects of androgen-deprivation therapy and fatigue. Additionally, the maximum number of children is 53, referring to the 53 genes in the ‘Gene’ class that are related to PCa.

**Table 1:** Summary of the PCAO2 structural parameters

Features	Count	Features	Count
No. of classes	633	Maximum number of children	53
No. of synonyms	2386	Average number of children	4
No. of object properties	33	Classes with a single child	20
Maximum depth	7	Classes with more than 25 children	2

**Figure 2:**
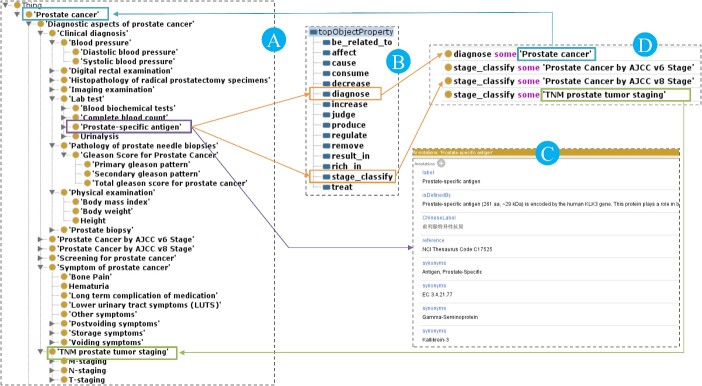
The upper-level classes, annotations and relationships of classes in the PCAO2 represented using the Protégé ontology editor. (**A**) is the ‘Prostate-specific antigen’ class in the PCa ontology hierarchical structure. (**B**) is the annotations of class ‘Prostate-specific antigen’. (**C**) and (**D**) show the object properties ‘diagnose’ and ‘stage_classify’ were used to link classes.

In PCAO2, each concept is accompanied by a scientific definition, synonyms, references and PMID. These concepts were initially collected manually from online resources such as the NCI Thesaurus, SNOMED-CT and MeSH. After comparison, we found 269 common concepts between PCAO2 and the NCI Thesaurus, 315 common concepts between PCAO2 and MeSH, 163 common concepts between PCAO2 and SNOMED CT and 4 common concepts between PCAO2 and FMA, respectively. If a concept was not present in these resources, we conducted searches in PCa guidelines, journal articles and other relevant sources. Figure 2 illustrates the annotations and logical relationships of the classes in PCAO2 using the Protégé ontology editor.

**Figure 3:**
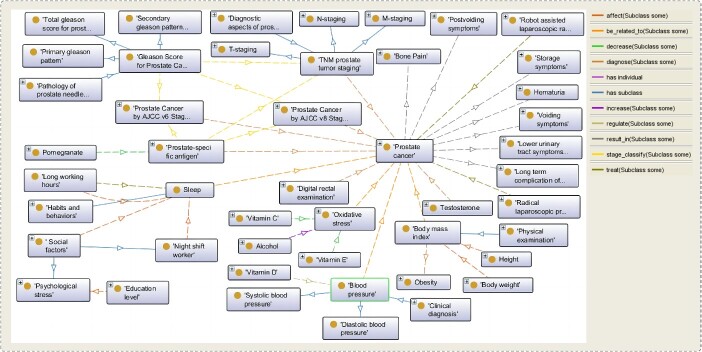
A part of the relationships between classes in the PCAO2 represented using the Protégé ontology editor. The rectangles with terms represent the classes in the ontology; the arrows represent the relationships between classes and different colors represent different object properties.

**Figure 4:**
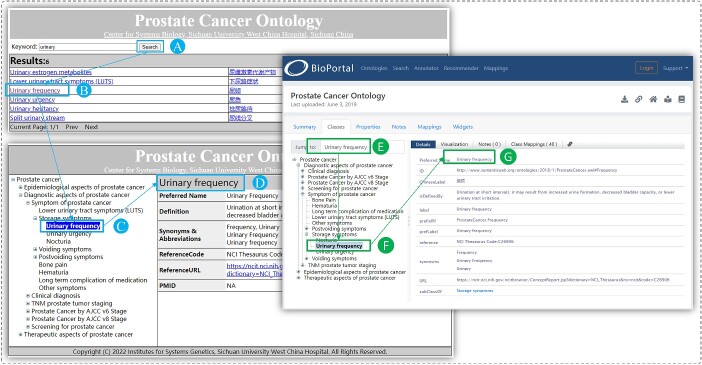
The PCAO2 information retrieval system. (**A**–**D**) and (**E**–**G**) show the query steps in PCAO information retrieval system we developed and the BioPortal website.

In PCAO2, object properties are used to establish links between classes, while datatype properties are used to connect classes with data values. For example, diagnostic indicators such as PSA values, Gleason score, DRE, prostate biopsy, pre-operative pathological diagnosis and TURP can be utilized for assessing the primary and secondary grades of PCa. In PCAO2, we employ the object property ‘diagnose’ to link these indicators, as shown in Figure 3. If clinically localized PCa patient’s TNM staging is T1c for Primary Tumor(T), Gleason score ≤ 6, PSA < 10 ng/ml, fewer than 3 prostate biospy cores positive and ≤50% cancer in each core, PSA density < 0.15 ng/ml/g, the patient can be grouped into the very low risk group. In PCAO2, we utilize the object property ‘group_in’ to associate individuals with their diagnostic values to specific risk groups. For example, in the lifestyle domain, consuming more ‘lamb meat’ within the ‘red meat’ category is associated with an increased risk of PCa. In PCAO2, we use the object property ‘increase_risk’ to link individuals who consume lamb meat with PCa. Figure 4 provides a thumbnail view of the relationships between the classes within PCAO2.

Compared to PCAO version 1.0 (PCAO1) published in 2009 [10], PCAO2 introduces several significant changes and improvements (Table 2). Firstly, PCAO1 primarily focuses on clinical data, while PCAO2 encompasses a broader range of data types, including genotypic, phenotypic and lifestyle data, allowing for a more comprehensive understanding of PCa. Secondly, PCAO2 expands its conceptual scope to include 633 concepts organized under three biomedical viewpoints, whereas PCAO1 addresses core clinical aspects with 412 concepts. Thirdly, PCAO2 incorporates a wider range of data sources, enhancing the richness and accuracy of the knowledge contained within the ontology. Lastly, PCAO2 integrates the previously developed PCLiON to standardize lifestyle data, which is not present in PCAO1. Additionally, PCAO2 includes genotypic information and gene associations, expanding the genomic aspect of PCa.

**Table 2:** Comparison of PCAO1 and PCAO2

Items	PCAO1	PCAO2
Data types	Clinical data	Genotypic, phenotypic and lifestyle data
Data source	NCI Thesaurus and FMA	Existed ontologies, clinical guidelines, NCI Thesaurus, SNOMED CT, MeSH, OMIM, ICD, DO, FMA, GTR, online books, PubMed, and Google
Concepts	412 concepts	633 concepts
Lifestyle data	Not available	Available
Genotypic data	Not available	Available
Accessibility	Not open source	Open source
System	Not available	Ontology-based system

### Information retrieval system

To enhance the usability of PCAO2, we have developed an online information retrieval system for PCAO2. Figure 4 shows a snapshot of the PCAO2 information retrieval system. The query keywords can be in English or Chinese, and a fuzzy search method allows for the retrieval of all records containing the input keywords.

The query interface consists of two columns: the first column displays links for English concepts, while the second column shows links for the corresponding Chinese concepts. By clicking on the different language links, the selected language version is displayed in the detailed information interface. If the query result spans multiple pages, paging function at the bottom of the results page enables page switching.

In the detail information interface, the left column displays the tree structure of the PCAO2, which is generated using a recursive algorithm (Supplementary Text S4). When a concept is clicked on in the query interface, it is selected in the tree structure. The right column shows annotations for the selected concept, including the preferred name, definition, synonyms, reference code, reference URL and PMID. If the selected node is changed in the tree view, the detail information will be refreshed to display the details of the newly selected node. The PCAO2 data are stored in a data table within the retrieval system. The definitions of the fields are listed in Table 3.

**Table 3:** Fields in the PCAO2 data table

Field name	Description	Example
id	Identity of each record	259
itemEN	English name of the concept	PSA velocity
itemCH	Chinese name of the concept	PSA速率
parentId	Identity of the parent node	256
preferredName	Preferred name of the concept	PSA velocity
definition	Definition of the concept	Measurement of how fast the PSA levels in the blood increase over time. A high PSA velocity may be a sign of prostate cancer and may help to find fast-growing prostate cancers.
synonyms	Synonyms of the concept	PSA velocity PSA Velocity PSA velocity
referenceCode	Reference code for other resources	NCI Thesaurus Code: C20119
referenceURL	Reference URL for other resources	https://ncit.nci.nih.gov/ncitbrowser/ConceptReport.jsp?dictionary=NCI_Thesaurus&ns=ncit&code=C20119
PMID	Reference PubMed paper ID	24578866, 22712027, 15714973

### Mobile intelligent terminal system

**Figure 5:**
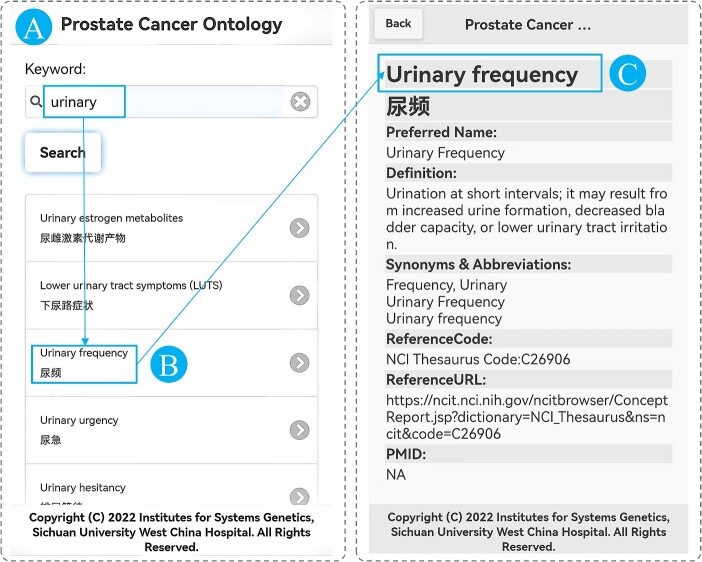
The mobile intelligent terminal system for the PCAO2. (**A**) is the query interface, (**B**) is the item interface and (**C**) is the detail interface.

Mobile intelligent terminals, such as smartphones and tablets, have been widely applied in recent years. In order to enable access to PCAO2 via mobile intelligent terminals, an online information system was developed. The access address for this system is the same as that for the information retrieval system. The program automatically redirects the client to the corresponding system. The mobile interfaces are shown in Figure 5, showcasing two interfaces denoted as A (query interface) and B (detail interface). In the query interface, when a user inputs keywords and clicks the search button, the results list will be displayed below the query button. To facilitate easy viewing, the results are sorted based on English concepts. The concepts in the result records are presented in both English and Chinese. When a user clicks on one of the results, the details of the concept are shown in the detail interface. Two data interfaces were implemented in the information retrieval system to facilitate data provision to the mobile intelligent terminal system (Supplementary Text S5).

### Scenario 1: Ontology-based PCa diagnosis and treatment data platform

PCaTreP is a data collection system that focuses on precision medicine and gathers diagnosis, treatment and follow-up information of PCa patients. It integrates various factors associated with PCa, such as pathogenic genes, lifestyle and environmental factors. The terminology used in PCaTreP is based on PCAO2. For instance, prostate-specific antigen (PSA) plays a significant role in the early diagnosis of asymptomatic PCa. Typically, a PSA level below 4.0 ng/ml is considered normal, while a PSA level higher than 10 ng/ml indicates an increased risk of PCa. The severity of tumor malignancy correlates with the extent of damage to normal prostate tissue, resulting in higher PSA levels in the serum.

**Figure 6:**
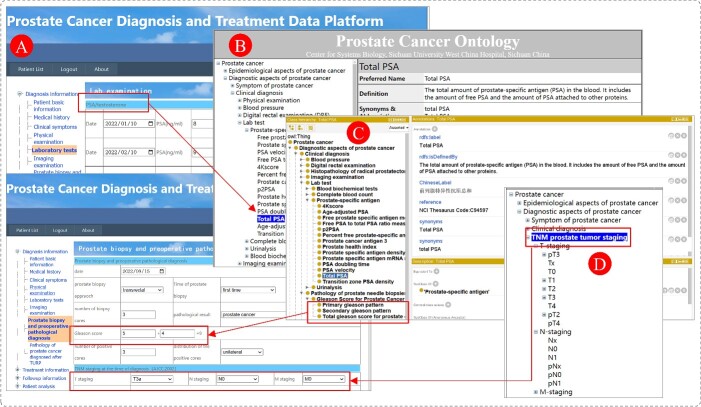
The relation between PCAO2 and PCaTreP. (**A**) is the PCaTreP, (**B**) is the PCAO2 online system, (**C**) is the ontology using Protégé Editor and (**D**) is the data source from PCAO2 for PCaTreP. PCAO2 is the data and relationship sources of PCaTrep.

Using the PCAO2-driven PCaTreP platform, clinicians can classify clinically localized PCa patients into risk groups based on the data collected in PCaTreP and relationships between the classes in PCAO2. For example, in PCaTreP, ‘PSA/Testosterone’ under ‘Laboratory tests’ collects the PSA test values, ‘Digital rectal examination’ under ‘Physical examinations’ records the results of DRE examinations and ‘Gleason score’ and ‘TNM grading’ are collected under ‘Prostate biopsy and preoperative pathological diagnosis’. By leveraging this data and the relationships within PCAO2, clinicians can efficiently and precisely devise treatment strategies for patients, taking into account their risk groups and expected survival time. Figure 6 presents a snapshot of PCaTreP based on PCAO2.

### Scenario 2: Ontology-driven precise improvement of PCa-related lifestyles

**Figure 7:**
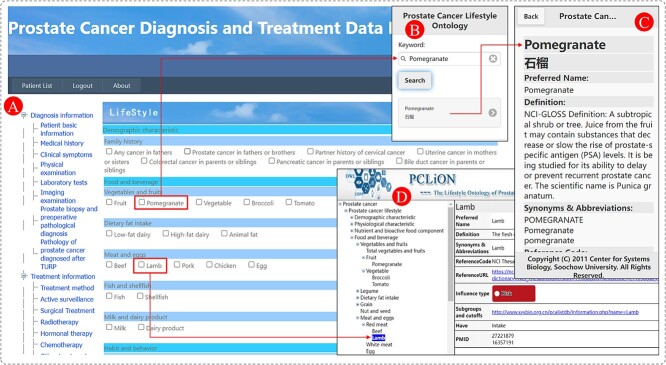
The relationship between PCLiON and PCaTreP. (**A**) is the PCaTreP, (**B**–**D**) are the PCLiON online system.

PCLiON ontology is a branch of the PCAO2. With PCLiON, we have integrated lifestyle data collection and improvement suggestion functions into PCaTreP. Figure 7 illustrates the relationship between PCLiON and PCaTreP. By analyzing the information provided by medical staff and referring to the identified risk factors and protective factors within the ontology, the system converts complex lifestyle research into accessible information for PCa patients. This information aims to guide patients in implementing accurate improvements.

For example, lamb meat, categorized under ‘Red meat’, is considered a risk factor. Compared to individuals who consume less lamb meat, those who consume higher quantities on a daily basis face an increased risk of developing PCa. On the other hand, grape seed oil, found in the ‘Health food’ category, acts as a protective factor. Regular consumption of an appropriate amount of grape seed oil can help prevent the onset of PCa or slow down its progression.

## DISCUSSION

Vast volumes of biomedical data have been generated as a result of the rapid development of sequencing technology and medical information systems. In the era of big data, domain ontologies have already demonstrated beneficial applications in text knowledge mining [9], heterogeneous data integration [10] and domain knowledge representation [11]. PCa ranks second among all malignant tumors affecting men worldwide. A significant amount of research data related to PCa has been accumulated from bench to bedside. However, an official ontology for PCa has not been previously published. In our previous study, Min introduced a method for integrating two databases at the semantic level using D2R-related technologies [10]. The study mentioned that the PCAO1 was constructed by merging PCa-related concepts from the NCI Thesaurus and FMA. A sample hierarchical structure was presented for therapeutic procedures, comprising 17 concepts. However, the PCAO1 was not described in detail, and the OWL file was not provided. Therefore, it was necessary to develop an ontology that encompasses knowledge from molecular biology to the clinical for PCa, as it is crucial for PCa-related data standardization, heterogeneous data integration and knowledge representation. To the best of our knowledge, PCAO2 is the earliest and relatively most comprehensive cancer ontology published on BioPortal. We have been studying PCAO2 since 2017 and uploaded it to BioPortal in 2019. Since then, we have continuously optimized the PCAO2. This ontology is important because it provides the foundation for personalized diagnosis and treatment of PCa.

Compared to PCAO1, PCAO2 expands the number of concepts to 633 organized under three biomedical viewpoints: epidemiology, diagnosis and treatment. This broader coverage enables a more comprehensive representation of the diverse aspects of PCa, and with significant overlaps observed with various biomedical ontologies and terminologies. For example, PCAO2 has 269 overlapping concepts with the NCI Thesaurus, 163 overlapping concepts with SNOMED CT, 91 overlapping concepts with GO, 23 overlapping concepts with DO and 9 overlapping concepts with ICD10-Clinical Modification.

The PCAO2 encompasses innovative lifestyle data related to PCa. PCa-related lifestyles were collected, organized and classified into the PCAO2 based on systematic evidence-based analysis. In subsequent studies, we plan to qualitatively analyze the relationship between lifestyles and PCa. This analysis holds significant importance for early personalized PCa prevention and the discovery of new diagnostic targets [42]. In order to improve the usability of PCAO2, we have developed an online information retrieval system. Recognizing the popularity of mobile intelligent terminals, we utilized HTML5, jQuery, jQuery Mobile, ASP.net and MySQL to implement a cross-platform mobile intelligent terminal system. Furthermore, to enable other applications to access knowledge from PCAO2, we have provided open access data interfaces. By utilizing the data interface protocols introduced in this study, applications can easily extract the necessary knowledge from PCAO2. Additionally, we have developed PCaTreP, a diagnosis and treatment data platform based on PCa domain knowledge standards. PCaTreP integrates multiple related systems such as diagnosis, treatment and prognosis of PCa under the new medical paradigm of precision medicine. PCLiON ontology is a specific branch of PCa ontology. Through the utilization of PCLiON ontology, we have incorporated lifestyle data collection and improvement suggestion functions into PCaTreP.

We referred to a large amount of PCa-related knowledge sources to construct the PCAO2. The advantages of PCAO2 include the following: (1) the concepts have been revised by PCa experts from reputable institutions such as the Urology Departments of the Second Affiliated Hospital of Soochow University and Sichuan University West China Hospital, etc., (2) PCAO2 includes information on PCa-related lifestyles and (3) PCAO2 contains the most comprehensive knowledge available in the field of PCa at present. PCAO2 also has some limitations: (1) the concepts related to PCa prevention, precision diagnosis and treatment exhibit diversity. (2) The definitions of the concepts need to be future standardized. (3) The list of synonyms in PCAO2 is not comprehensive and complete.

It is important to recognize that the development of an ontology is an iterative process. In our future research, we will continuously update PCAO2 to address the issues identified through practical applications. First, our primary focus will be on advancing ontology standardization. To ensure consistent and meaningful exchange of information, PCAO2 will provide a standardized ontology. To further enhance this standardization, future efforts will concentrate on aligning PCAO2 with other ontological frameworks and industry standards, such as HL7. This alignment will promote interoperability and facilitate data exchange across different healthcare systems. Second, we will expand the concept coverage of PCAO2. By actively collaborating with domain experts, clinicians and researchers, PCAO2 will continuously incorporate emerging knowledge, biomarkers, therapies, as well as patient perspectives and experiences. Third, PCAO2 will integrate with other areas, including genetic variations, molecular pathways, imaging findings and electronic medical records. By providing a multidimensional representation, PCAO2 will facilitate personalized medicine and enable the development of advanced analytics and decision support systems. Lastly, we recognize the potential of utilizing ontology-based knowledge architecture and logical frameworks in the era of large language models and AI. By integrating ontology-based knowledge structures with advanced machine learning techniques, we can design algorithmic approaches for prompt learning. This integration will further enhance the interpretability and transparency of the system.

## CONCLUSION

The PCAO2 aims to collect and organize knowledge of PCa from various perspectives, including epidemiology, diagnosis and treatment. Its goal is to provide a systematic, formalized, structured and computer-readable knowledge framework. Currently, the PCAO2 is at version 2.0, and we are planning to establish an alliance and invite more international experts to participate in the development of version 3.0. PCAO2 serves as a valuable reference for the development of other cancer ontologies and plays a crucial role in driving advancements in this field. Furthermore, we anticipate that the scientific community, experts and researchers will contribute valuable suggestions and advice to further enhance future versions of PCAO2.

**Key Points**

- We have developed an updated version of the Prostate Cancer Ontology (PCAO2), which collects and organizes knowledge of prostate cancer (PCa) from genotypic, phenotypic and lifestyle data and is organized under three biomedical viewpoints, namely, epidemiology, diagnosis and treatment.
- PCAO2 includes scientific definitions, synonyms, relationships and references for each concept and provides a standardized and systematized semantic framework for studying heterogeneous PCa data and knowledge.
- We have developed an information retrieval system and a mobile interface for PCAO2, which provide a tree hierarchical structure view and enhance usability and accessibility.

## Supplementary Material

supplementary_data_bbae136

## Data Availability

The PCAO2 is freely available at https://bioportal.bioontology.org/ontologies/PCAO, http://pcaontology.net/ and http://pcaontology.net/mobile/.

## Author Biographies

**Chunjiang Yu** is a professor at School of Artificial Intelligence, Suzhou Industrial Park Institute of Services Outsourcing. His research interest is biomedical informatics, genetics and medical systems biology.

**Hui Zong** is a postdoctoral researcher at Institutes for Systems Genetics, West China Hospital, Sichuan University. His research interest is biomedical informatics, digital health and natural language processing.

**Yalan Chen** is an associate professor at Department of Medical Informatics, School of Medicine, Nantong University. Her research interest is disease-related lifestyle and health management.

**Yibin Zhou** is a clinician at Department of Urology, The Second Affiliated Hospital of Soochow University. His research interest is prostate cancer.

**Xingyun Liu** is a researcher at Institutes for Systems Genetics, West China Hospital, Sichuan University. His research interest is biomarker, database and ontology.

**Yuxin Lin** is a researcher at Department of Urology, The First Affiliated Hospital of Soochow University. His research interest is prostate cancer.

**Jiakun Li** is a postdoctoral researcher at Department of Urology and Institutes for Systems Genetics, West China Hospital, Sichuan University. His research interest is prostate cancer, bioinformatics and transformation of prostate cancer.

**Xiaonan Zheng** is a postdoctoral researcher at Department of Urology and Institutes for Systems Genetics, West China Hospital, Sichuan University. His research interest is urological malignant tumors.

**Hua Min** is an associate professor at Department of Health Administration and Policy, George Mason University. Her research interest is health informatics, medical standards and ontologies.

**Bairong Shen** is a professor and executive director at Institutes for Systems Genetics, West China Hospital, Sichuan University. His research interest is biomedical informatics, genetics and medical systems biology.
