# Supplementary material for: PCAO2: an ontology for integration of prostate cancer associated genotypic, phenotypic and lifestyle data
Source: Brief Bioinform. 2024 Mar 31;25(3):bbae136. doi: 10.1093/bib/bbae136 (PMC10982949; doi:10.1093/bib/bbae136)
Supplement: supplementary_data_bbae136 [file supplementary_data_bbae136.docx]

**PCAO2: an ontology for integration of prostate cancer associated genotypic, phenotypic and lifestyle data**

Chunjiang Yu^1,2,3,†^, Hui Zong^1,†^, Yalan Chen^1,3,4,†^, Yibin Zhou^5^, Xingyun Liu^1^, Yuxin Lin^6^, Jiakun Li^1^, Xiaonan Zheng^1^, Hua Min^7,*^, Bairong Shen^1,*^

1. Department of Urology and Institutes for Systems Genetics, Frontiers Science Center for Disease-related Molecular Network, West China Hospital, Sichuan University, Chengdu, 610041, China.

2. School of Artificial Intelligence, Suzhou Industrial Park Institute of Services Outsourcing, Suzhou, 215123, China

3. Center for Systems Biology, Soochow University, Suzhou, 215006, China

4. Department of Medical Informatics, School of Medicine, Nantong University, Nantong, 226001, China

5. Department of Urology, The Second Affiliated Hospital of Soochow University, Suzhou, 215011, China

6. Department of Urology, The First Affiliated Hospital of Soochow University, Suzhou, 215000, China

7. Department of Health Administration and Policy, George Mason University, Fairfax, VA, USA

† These authors contributed equally

* To whom correspondence should be addressed:

Bairong Shen, PhD, Professor, Institutes for Systems Genetics, Frontiers Science Center for Disease-related Molecular Network, West China Hospital of Sichuan University, No. 37 Guoxue Alley, Chengdu, Sichuan, China; Tel: 86-15995854635; Email: bairong.shen@scu.edu.cn

Hua Min, PhD, Department of Health Administration and Policy, George Mason University, Fairfax, VA, USA; Email: hmin3@gmu.edu

**Supplementary: Text**

**S1: Expert panels**

Experts from various institutions, including the Urology Department of Sichuan University West China Hospital, the Department of Health Administration and Policy of George Mason University, the Urology Department of Suzhou University Affiliated Second Hospital, the Laboratory Department of Nantong University Affiliated Hospital, and the School of Public Health of Nantong University, actively participated the study.

The establishment of the overall framework was gradually formed through team meetings, and experts from relevant fields were invited for each part. For the diagnosis and treatment part, doctors from the Urology Department of Suzhou University Affiliated Second Hospital and the Laboratory Department of Nantong University Affiliated Hospital were participated in. For the genetic part, experts from the West China Institute of Genetics were participated in. For the imaging part, doctors from the imaging department of Shanghai Huashan Hospital we participated in. For the lifestyle part, we collected literature and asked professors from the School of Public Health of Nantong University to conduct a thorough review word by word. The overall principle is to collect and review according to different fields and categories.

**S2: System development and applications**

The mobile intelligent terminal system was developed using HTML5, jQuery, and jQuery Mobile. jQuery is a high-speed, concise JavaScript framework that uses the permissive MIT license. In this system, the Ajax technology comprising jQuery was used to call data interfaces from the server and exchange data between the intelligent terminal and server. jQuery Mobile, built on jQuery and the jQuery UI class library, provided a front-end framework for creating mobile web applications. By utilizing jQuery Mobile, developers can accomplish more with less code. It allows for flexible and simple organization of web pages and is compatible with all mobile devices. We utilized the jQuery Mobile layout framework and controls to design the interfaces. To display the results, jQuery was used to write the data received from the server to the jQuery Mobile controls. JSON is a lightweight data interchange format, where the text format employed is completely independent of the programming language used for storing and representing data. The JSON format is easy for researchers to read and write, and it is also easy to parse and generate in hardware. The JSON format effectively improves network transmission efficiency. We used the JSON data format to exchange data between mobile intelligent terminals and the server.

**S3: The epidemiological, diagnostic and therapeutic aspects in PCAO2**

The epidemiological aspects viewpoint of PCAO2 describes basic information, personal history, and lifestyles of patients. Basic information encompasses age, occupation, race, etc. Personal history includes details on hypertension, diabetes mellitus, hyperglycemia, etc. The lifestyle incorporates demographic characteristics, habits, environmental factors, etc. Although only a limited number of studies have explored the connections between lifestyle and specific diseases, we conducted a comprehensive search in PubMed for reports on lifestyles and environmental factors associated with PCa. The search terms consisted of "prostate cancer," "risk factor," "lifestyle," "vitamin," "smoke*," "wine," as well as "tea," "coffee," "diet," "dairy," "social," or "environment*." All relevant factors, such as diet, habits, diseases associated with habits, and drugs were collected and included in the lifestyle viewpoint for PCa. Currently, there is no standardized classification for lifestyles associated with a specific disease. Therefore, to develop a lifestyle ontology for prostate cancer (PCa), we initially categorized all potential lifestyles relevant to PCa. We constructed a general classification framework for PCa-related lifestyles based on a study conducted by Cuzick ^1^. Subsequently, we iteratively expanded and enhanced the framework in a follow-up study. After completing the preliminary classification framework, we consulted relevant experts and added second and third subgroups to the classification, as shown in Figure 3. We also referred to the Third Expert Report of the WCRF/AICR ^2^. In the section on PCa, we obtained details of qualitative analyses of the lifestyle factors associated with PCa ^3^. Consequently, additional lifestyle factors were qualitatively analyzed to determine their attributes in relation to PCAO2. Furthermore, we extracted genes from the Genetic Testing Registry website ^4^, and currently, 65 genes are included in the PCAO2 dataset. Furthermore, we extracted genes from the Genetic Testing Registry website ^4^, and currently, 65 genes are included in the PCAO2 dataset.

The diagnostic aspects viewpoint of PCAO2 describes the symptoms, clinical diagnosis, and TNM staging, etc. PCAO2 symptoms encompass lower urinary tract symptoms, storage symptoms, voiding symptoms, etc. Clinical diagnosis involves physical examination, laboratory examination, digital rectal examination, etc. TNM staging for PCa includes T-staging, N-staging, M-staging, and so on.

The therapeutic aspects viewpoint of PCAO2 covers deferred treatments, surgery, radiotherapy, hormonal therapy, chemotherapy, and others. Deferred treatments consist of active surveillance and watchful waiting. Surgery includes open prostatectomy, radical laparoscopic prostatectomy, robot-assisted laparoscopic radical prostatectomy, and more. Radiotherapy encompasses external beam radiation therapy, proton beam therapy, brachytherapy, and others. Hormonal therapy includes castration, anti-androgens, testosterone-lowering therapy, etc. Chemotherapy includes adriamycin, cabazitaxel, cisplatin, etc.

**S4: Construction of tree structure**

In the detail information interface, the tree structure of PCAO2 is generated using a recursive algorithm. Initially, the root node is created to establish the foundation of the tree structure. Then, program searches the database to find all child nodes of the root node. We have developed a recursive method with parameters consisting of the root node and a list of its child nodes. This recursive method returns null if the root node has no child nodes. However, if the root node does have child nodes, the following loop operations are performed for each child node. In summary, the recursive method involves three steps: First, a child node is created and added to the newly created node for the root node. Second, the method searches for nodes in the database where the parent node is the newly created node. Third, the recursive method is called, with the newly created node and all its child nodes as parameters.

**S5: Data query interface and detail information interface**

The data query interface and detail information interface were developed to provide data to the mobile intelligent terminal system. These two interfaces are open and they can be called by other applications. The website link of the data query interface is at *http://pcaontology.net/PCaSearchInterface.aspx?keyword=[input_kw]*. *[input_kw]* in the address is the query keyword. The keyword can be in English or Chinese. The values returned to the interfaces use the JavaScript Object Notation (JSON) format. A fuzzy query technique is used to present the query keyword with the concepts from the PCAO2. When the data interface receives the keyword, the following processes are conducted. First, the query statement is created. Second, the query statement is used to search the database. Finally, the search results are converted into the JSON format and returned to the program, which calls the data interface. For example, if “PSA Velocity” or “PSA速率” is used as the keyword, the query result is as follows.

*[“id”: 259, “itemCH”: “PSA速率,” “itemEN”: “PSA Velocity” ].*

The meanings of these fields are explained in Table 2. If the results contain multiple records, then more than one ‘content’ in *‘[]’* is separated by commas, e.g., [content-1, content-2, …, content-n].

The website link of the detail information interface is at *http://pcaontology.net/PCaDetailInterface.aspx?id=[input_id]*. *[input_id]* in the address is the identity of the record that needs to be retrieved. For example, if the *[input_id]* is 259, then the retrieval results are as follows.

*[“id”: 259, “itemCH”: “PSA速率,” “itemEN”: “PSA Velocity,” “parentId”: 256, “PreferredName”: “PSA Velocity,” “Definition”: “A measurement of how fast PSA levels in the blood increase over time. A high PSA velocity may be a sign of prostate cancer and may help to find fast-growing prostate cancers.|,” “SynonymsAbbreviations”: “Prostate Specific Antigen Velocity |PSA Velocity |PSA velocity,” “ReferenceCode”: “NCI Thesaurus Code:C20119,” “ReferenceURL”: “https://ncit.nci.nih.gov/ncitbrowser/ConceptReport.jsp?dictionary=NCI_Thesaurus&ns=ncit&code=C20119,” “PMID”: “NA” ].*

**Reference**

1. Cuzick, J., Thorat, M.A., Andriole, G., Brawley, O.W., Brown, P.H., Culig, Z., Eeles, R.A., Ford, L.G., Hamdy, F.C., Holmberg, L., et al. (2014). Prevention and early detection of prostate cancer. Lancet Oncol *15*, e484-492. 10.1016/S1470-2045(14)70211-6.

2. Ballon-Landa, E., and Parsons, J.K. (2018). Nutrition, physical activity, and lifestyle factors in prostate cancer prevention. Curr Opin Urol *28*, 55-61. 10.1097/MOU.0000000000000460.

3. Chen, Y., Yu, C., Liu, X., Xi, T., Xu, G., Sun, Y., Zhu, F., and Shen, B. (2021). PCLiON: An Ontology for Data Standardization and Sharing of Prostate Cancer Associated Lifestyles. Int J Med Inform *145*, 104332. 10.1016/j.ijmedinf.2020.104332.

4. Rubinstein, W.S., Maglott, D.R., Lee, J.M., Kattman, B.L., Malheiro, A.J., Ovetsky, M., Hem, V., Gorelenkov, V., Song, G., Wallin, C., et al. (2013). The NIH genetic testing registry: a new, centralized database of genetic tests to enable access to comprehensive information and improve transparency. Nucleic Acids Res *41*, D925-935. 10.1093/nar/gks1173.

**Supplementary: Figure**


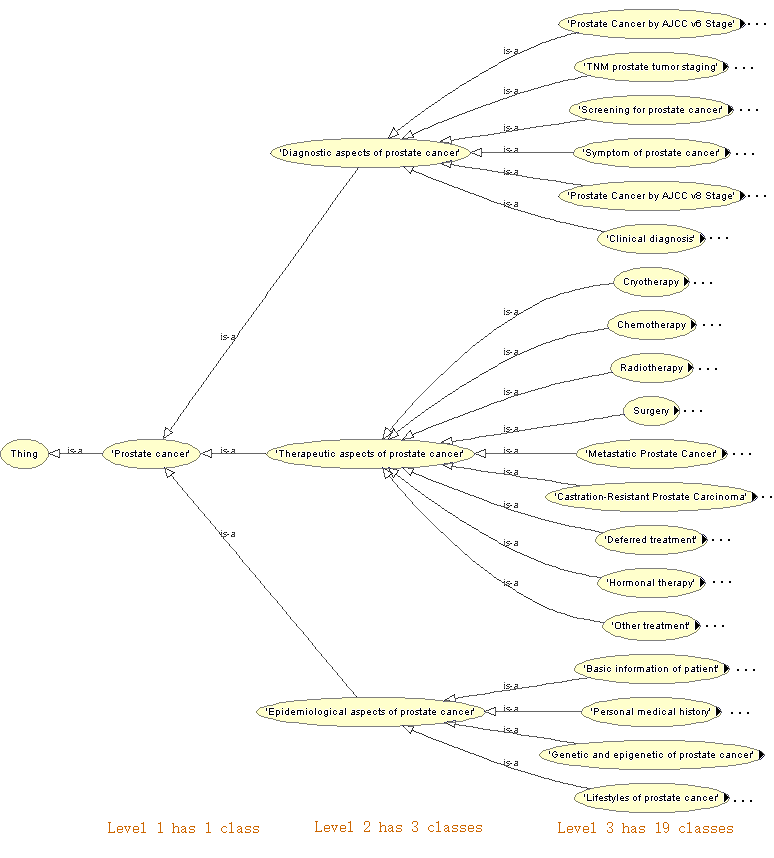


**Figure S1.** The upper three level of relationship *is_a* between classes.
